# Percutaneous ultrasound-guided drainage of pneumomediastinum through the retropharyngeal space: a case report

**DOI:** 10.1093/gastro/goac040

**Published:** 2022-08-11

**Authors:** Si Qin, Xu-Tao Lin, Yi-Min Wang, Yao Chen, Rui Cui, Guang-Jian Liu

**Affiliations:** Department of Medical Ultrasonics, The Sixth Affiliated Hospital, Sun Yat-sen University, Guangzhou, Guangdong, P. R. China; Department of Gastrointestinal Endoscopy, The Sixth Affiliated Hospital, Sun Yat-sen University, Guangzhou, Guangdong, P. R. China; Guangdong Provincial Key Laboratory of Colorectal and Pelvic Floor Diseases, The Sixth Affiliated Hospital, Sun Yat-sen University, Guangzhou, Guangdong, P. R. China; Department of Medical Ultrasonics, The Sixth Affiliated Hospital, Sun Yat-sen University, Guangzhou, Guangdong, P. R. China; Department of Medical Ultrasonics, The Sixth Affiliated Hospital, Sun Yat-sen University, Guangzhou, Guangdong, P. R. China; Department of Medical Ultrasonics, The Sixth Affiliated Hospital, Sun Yat-sen University, Guangzhou, Guangdong, P. R. China; Department of Medical Ultrasonics, The Sixth Affiliated Hospital, Sun Yat-sen University, Guangzhou, Guangdong, P. R. China

## Introduction

Esophageal perforation (EP) is a rare but potentially lethal clinical condition with a mortality rate as high as 20% [1]. The most common causes of EP are iatrogenic, spontaneous, and foreign body ingestion [2]. EP is often accompanied by serious complications such as hemorrhagic pericardial effusion, mediastinal abscesses, and sepsis [3]. Management is multidisciplinary and involves emergency physicians, thoracic surgeons, otaorhinolaryngologists, gastroenterologists, anesthesiologists, and radiologists [1]. We herein report a case of pneumomediastinum caused by EP and discuss the effect of percutaneous ultrasound (US)-guided drainage through the retropharyngeal space.

## Case report

A 74-year-old woman developed EP and mediastinal and thoracic infection after endoscopic submucosal dissection for esophageal low-grade squamous intraepithelial neoplasia. Emergency surgery including thoracoscopic exploration and endoscopic esophageal wound closure was performed. Anti-infective treatment, proton-pump inhibitor therapy, and other symptomatic treatments were given post-operatively. However, the patient’s condition became unstable within 10 days post-operatively. She developed a fever (the highest temperature, 38.1°C) accompanied by a decreased oxygenation index, but her white cell count and procalcitonin concentration did not decrease. Eleven days post-operatively, chest computed tomography (CT) showed pneumomediastinum and retropharyngeal emphysema. After a multidisciplinary discussion, puncture and drainage were suggested considering that the infection source was still present without the conditions for reoperation.

To drain the pneumomediastinum, transthoracic drainage was blocked by the sternum, ribs, aorta, and lungs. According to the CT image, the pneumomediastinum extended up into the retropharyngeal space. Therefore, we considered puncture through the retropharyngeal space and catheterization into the mediastinum for drainage. A LOGIQ E9 US system (GE Healthcare, Chicago, IL, USA) equipped with a 9-L high-frequency linear probe was used for US examination. US showed retropharyngeal emphysema posterior to the thyroid. The liquid-isolating technique was performed to create a safe puncture path [4]. US guidance was used to inject 10 mL of 1% lidocaine to fill the space between the thyroid lateral capsule and the carotid artery as well as to establish local anesthesia. A liquid barrier was thus created to separate the thyroid gland from the carotid artery and internal jugular vein. With use of US guidance, an 18-gauge needle was passed in the craniocaudal direction through the skin and sternocleidomastoid muscle, through the liquid barrier between the carotid artery and thyroid, and into the gas cavity. The needle core was pulled out after feeling a sense of breakthrough. Gas was aspirated, indicating that the needle tip was located in the emphysema. With use of the Seldinger technique, a 12-F pigtail catheter was placed into the emphysema and connected to a negative-pressure suction device. Ten milliliters of pus were aspirated and sent for culture. A nasogastric tube was then endoscopically placed, and the nasogastric tube and mediastinal drainage tube formed a good hedge drainage.

**Figure 1.**
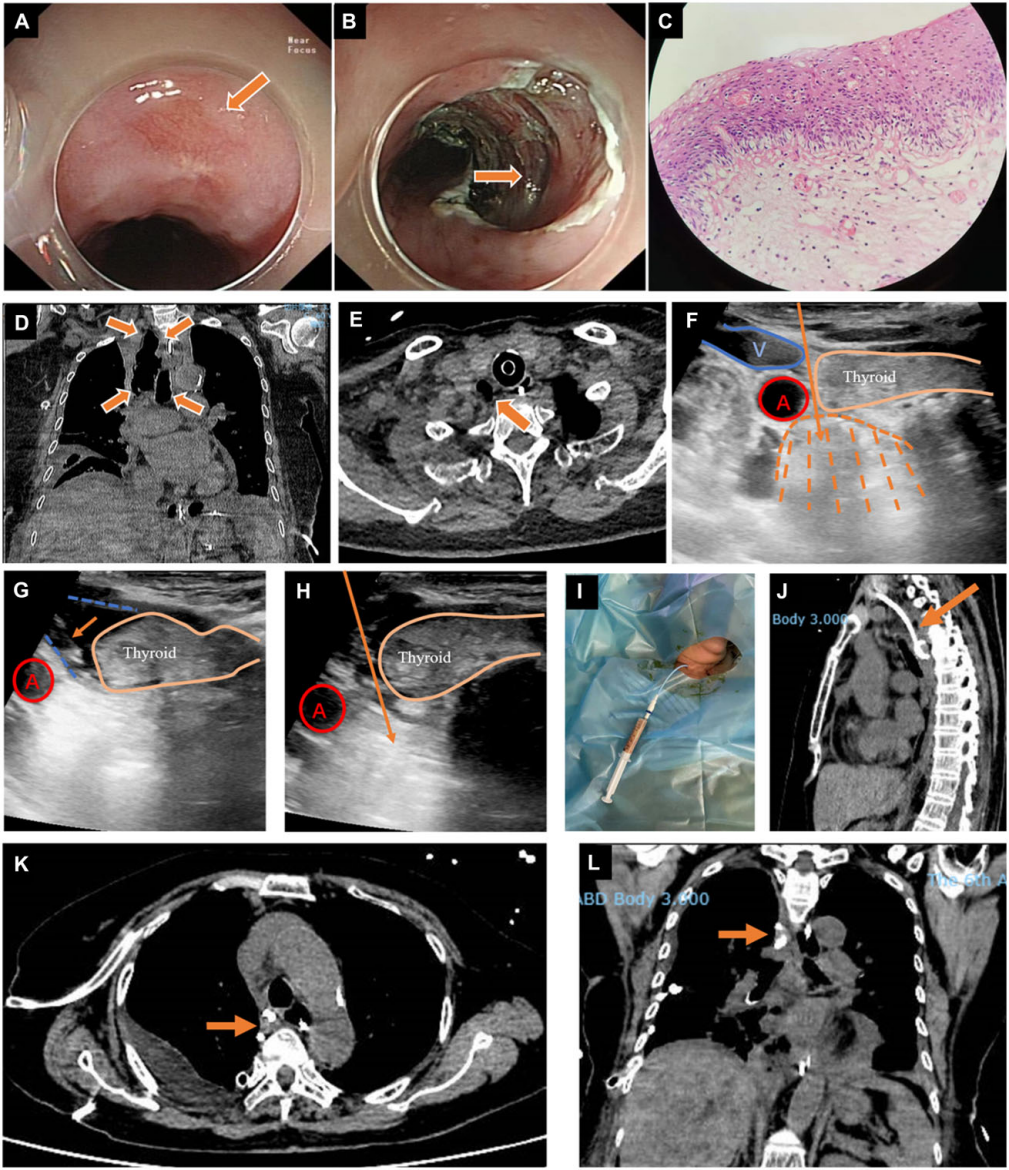
The clinical data of the patients. (A) Esophagoscopy showed lesions of the esophageal mucosa (thick arrow). (B) Endoscopic submucosal dissection was performed and part of the muscularis propria was damaged (thick arrow). (C) Microscopic image (hematoxylin–eosin staining; original magnification, ×20) of the specimen obtained by endoscopic submucosal dissection showed esophageal low-grade squamous intraepithelial neoplasia. Chest computed tomography 11 days after surgery for the esophageal perforation showed pneumomediastinum (thick arrows) (D) and retropharyngeal emphysema (thick arrow) (E). (F)–(I) Percutaneous ultrasound-guided drainage of the pneumomediastinum through the retropharyngeal space. (F) Ultrasound showed retropharyngeal emphysema posterior to the thyroid (dashed area) and the puncture path (thin arrow) was designed through the space between the thyroid lateral capsule and the carotid artery and internal jugular vein. (G) Ultrasound guidance was used to inject (thin arrow) 10 mL of 1% lidocaine to fill the space (dotted box) between the thyroid lateral capsule and the carotid artery as well as to provide local anesthesia. (H) With use of ultrasound guidance, an 18-gauge needle (thin arrow) was passed in the craniocaudal direction through the skin and sternocleidomastoid muscle, through the liquid barrier between the carotid artery and thyroid, and into the gas cavity. (I) With use of the Seldinger technique, a 12-F pigtail catheter was placed into the emphysema and 10 mL of pus were aspirated. (J)–(L) Chest computed tomography 6 days after ultrasound-guided drainage showed absorption of the pneumomediastinum and retropharyngeal emphysema; (J) sagittal plane; (K) cross section; (L) coronal plane; the arrows indicate the pigtail catheter. A, carotid artery; V, internal jugular vein.

Six days after US-guided drainage, the patient had no fever and her inflammatory indexes had decreased. Repeat chest CT showed absorption of the pneumomediastinum and retropharyngeal emphysema (Figure 1). Chest CT on post-operative Day 30 showed no evidence of residual perforation. The drainage catheter was removed after this study and the patient’s diet was advanced to regular. She was discharged home and completed a 2-week course of oral antibiotics. At the 1-month follow-up visit, the patient was doing well without complications.

## Discussion

Drainage of the mediastinum is part of aggressive minimally invasive management of EP [1, 5]. There are several options to drain mediastinal abscesses, including open surgery, mediastinoscopy, thoracoscopic surgery, endobronchial US-guided transbronchial needle aspiration, and image-guided transthoracic drainage. Each technique has advantages and disadvantages in terms of accuracy, invasion, cost, and risk [6]. Image-guided transthoracic drainage is less invasive and cost-effective than other alternative methods. CT is commonly used for percutaneous radiology technique guidance during puncture of the mediastinum [6]. The drawback is that CT guidance carries a significant amount of radiation exposure and has a risk of damaging important vessels and nerves or causing pneumothorax. Percutaneous US guidance is a rapid, convenient technique that permits real-time observation of the catheter without ionizing radiation exposure [7]. However, since US is easily blocked and impeded by bones, lungs, and gas, it is generally used for puncture of large lesions in the anterior mediastinum [6]. Our patient developed thoracic EP complicated by pneumomediastinum. Transthoracic drainage would have been blocked by the sternum, ribs, aorta, and lungs, resulting in difficult puncture. The mediastinal space extends upward with the cervical fascia and space. As a result, mediastinal infection may diffuse upward to the neck [8]. In our patient, the pneumomediastinum extended up into the retropharyngeal space, creating conditions for drainage of the pneumomediastinum through the retropharyngeal space.

The liquid-isolating technique, also known as hydrodissection, is widely used to protect vital tissue from thermal energy during image-guided ablation of thyroid nodules. The main idea of liquid isolation is to inject liquid (such as 0.9% saline) into the spaces between the thyroid capsule and the anterior cervical muscles, blood vessels, trachea, and esophagus, thereby creating a liquid barrier that protects sensitive tissue from excessive heat. With reference to the liquid-isolating technique commonly used in thyroid thermal ablation, a liquid barrier was established along the lateral side of the thyroid to push the thyroid away from the carotid artery and internal jugular vein, creating a safe path of puncture to the retropharyngeal space [4]. Because the puncture time is short, a large amount of fluid is not required. In our patient, 10 mL of 1% lidocaine was sufficient to establish a liquid barrier and provide local anesthesia. We treated the pneumomediastinum with percutaneous US-guided drainage through the retropharyngeal space to avoid surgical re-intervention and eliminate the additional risks associated with radiation of CT.

In conclusion, EP combined with mediastinal infection is a difficult problem that requires multidisciplinary treatment. Percutaneous US-guided drainage through the retropharyngeal space is a potentially effective minimally invasive treatment for pneumomediastinum and mediastinal abscesses in carefully selected patients, and the liquid-isolating technique helps to create a safe puncture path. Certainly, there are numerous circumstances in which this procedure may not be appropriate. CT and US images need to be combined to find a safe and effective puncture path.

## Funding

None.
